# Application of Semiautomatic Fetal Intelligent Navigation Echocardiography (FINE) in Twin Pregnancies: Half the Work or Twice the Effort?

**DOI:** 10.7759/cureus.38052

**Published:** 2023-04-24

**Authors:** Michael Gembicki, Amrei Welp, Jann Lennard Scharf, Christoph Dracopoulos, Jan Weichert

**Affiliations:** 1 Obstetrics and Gynaecology, Universitätsklinikum Schleswig-Holstein, Luebeck, DEU

**Keywords:** automatization, 3d/4d, ultrasound, stic, spatiotemporal image correlation, twin pregnancies, fetal echocardiography

## Abstract

Objective: To assess the performance of fetal intelligent navigation echocardiography (FINE, 5D Heart™) for automated volumetric investigation of the fetal heart in twin pregnancies.

Methods: Three hundred twenty-eight twin fetuses underwent fetal echocardiography in the second and third trimesters. Spatiotemporal image correlation (STIC) volumes were obtained for a volumetric investigation. The volumes were analyzed using the FINE software, and the data were investigated regarding image quality and many properly reconstructed planes.

Results: Three hundred and eight volumes underwent final analysis. 55.8% of the included pregnancies were dichorionic twin pregnancies, and 44.2% were monochorionic twin pregnancies. The mean gestational age (GA) was 22.1 weeks, and the mean maternal BMI was 27.3 kg/m^2^. The STIC-volume acquisition was successful in 100.0% and 95.5% of cases. The overall depiction rates of FINE were 96.5% (twin 1) and 94.7% (twin 2), respectively (p = 0.0849, not significant). In 95.9% (twin 1) and 93.9% (twin 2), at least 7 planes were reconstructed properly (p = 0.6056, not significant).

Conclusion: Our results indicate that the FINE technique used in twin pregnancies is reliable. No significant difference between the depiction rates of twin 1 and twin 2 could be detected. In addition, the depiction rates are as high as those derived from singleton pregnancies. Due to the challenges of fetal echocardiography in twin pregnancies (i.e., greater rates of cardiac anomaly and more difficult scans), the FINE technique might be a valuable tool to improve the quality of medical care in those pregnancies.

## Introduction

It is well known that congenital heart disease (CHD) is the most common organ-specific congenital disability, with a prevalence of 7-9 per 1,000 singleton live births [1-5]. CHD is the most important reason for neonatal morbidity and mortality from congenital disabilities [4,6]. One big challenge is caused by the fact that only about 10% of CHD cases occur in a high-risk population, which increases the difficulty of prenatal diagnosis of CHD in a low-risk population [4,7,8] because only in the minority of those cases is fetal echocardiography performed. Despite several efforts, the prenatal detection rates of CHD stayed relatively low, ranging from 15-39% [9,10]. There are multiple reasons why detecting CHD is difficult. The fetal heart moves fast, and the anatomy is small-scaled and complex [11]. In addition, external factors such as the maternal BMI and the investigator's experience make a difference, too [12].

Another critical point is the diagnostic challenge of fetal echocardiography in twin pregnancies. The targeted ultrasound screening in twin pregnancies may be more difficult than usual because of the presence of a second fetus, and it is important to allow more time for the scan [13]. Compared with singleton pregnancies, the risk of a fetal anomaly is greater in twins. CHDs are much more common in twin pregnancies, with a reported prevalence of up to 20 in 1000 live births [5]. The rates per fetus in dizygotic twins seem the same as that in singletons, whereas it is two-to-three times higher in monozygotic twins [14,15]. In monochorionic twins, the risk for CHD has been estimated at 2 to 9% [16]. Due to the relatively low prenatal detection rates and the additional challenges in twin pregnancies, technical approaches could help improve the quality of care. Four-dimensional (4D) ultrasound with spatiotemporal image correlation (STIC) has been demonstrated to overcome some of the mentioned difficulties [4,17-20] and has the potential to increase the detection rates of CHD [18,21,22]. Nevertheless, analyzing a STIC volume remains relatively difficult [11]. Most importantly, the investigator must have profound knowledge of fetal cardiac anatomy, especially in altered cases [11,23-25].

A semiautomatic algorithm including artificial intelligence (Fetal Intelligent Navigation Echocardiography, FINE, commercially known as 5D HeartTM) was invented to face the problems of working with STIC volumes [11]. This more operator-independent technique automatically displays all views [26,27] mandatory for a sophisticated investigation of the fetal heart [28,29]. There is no need to adjust the volumes manually [4].

FINE showed up to 100% of the nine fetal echocardiography views using STIC volumes of structurally inconspicuous fetal hearts [11,23,24,30]. In cases of CHD, FINE showed a sensitivity of 98% and a specificity of 93% [31]. Other groups have demonstrated that FINE works well in cases of fetal D-transposition of the great arteries [32] and works as well as traditional 2D fetal echocardiography with a significantly lower investigation time [33]. Our data indicated the applicability of FINE in different states of investigator experience [30] and in inconvenient fetal spine positions [34]. The most recent data show a good performance of FINE static mode [35], a technique based on very rapid acquisition time STIC volumes (e.g., 1s) and could be helpful in more active fetuses.

For proper results, FINE needs proper STIC volumes. The quality depends on several factors (e.g., fetal spine position, shadowing, fetal movement, and image clarity) [11]. As mentioned, fetal echocardiography may be more altered by these factors in twin pregnancies than in singleton pregnancies. Therefore, this study examines FINE's applicability and performance for semiautomatic volumetric assessment of the normal fetus in twin pregnancies.

Preliminary data of our study was presented at the American Institute of Ultrasound in Medicine (AIUM) UltraCon 2023 in Orlando, FL, USA.

## Materials and methods

Subjects

All women presenting for a fetal ultrasound at our department undergo additional 3D and 4D fetal echocardiography with STIC volume acquisition besides conventional 2D examination. Data were acquired between June 2016 and March 2023. For the current study, we have included twin pregnancies older than 13+6 weeks of gestation regardless of chorionicity and maternal factors (e.g., BMI). Structurally abnormal fetuses were excluded, whereas cases of twin-to-twin transfusion syndrome (TTTS) were not. Expert investigators performed all investigations. The recorded volumes were of specific quality (e.g., minimal or absent shadowing; a visible transverse aortic arch; minimal fetal movement, and adequate image clarity).

Acquisition of STIC volumes

The volumes were recorded and investigated by two physicians, both being experts in fetal echocardiography (JW and MG), using a GE Voluson E10 (GE Healthcare, Chicago, United States of America), a Samsung WS80A and a Samsung Hera W10 (Samsung Medison, Seoul, Korea). All machines were equipped with mechanical convex transducers. The acquisition time ranged from 9 to 12s, and the acquisition angle ranged from 15 and 35°, depending on gestational age.

Examination with FINE

**Figure 1:**
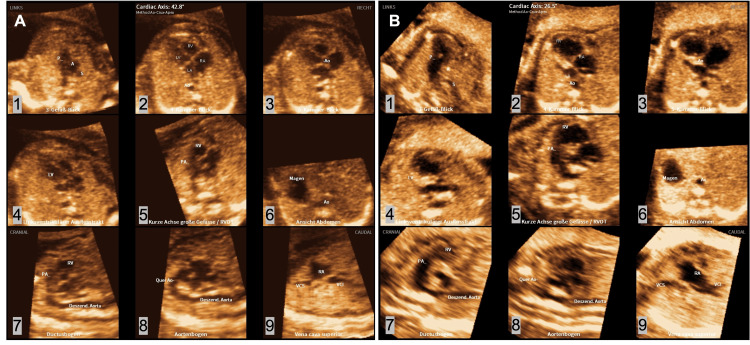
All views of the structurally normal (A) (normal heart, a 21-year-old female patient at 17+1 weeks of gestation, BMI 25.4 kg/m^2) and abnormal (B) (hypoplastic left heart, a 34-year-old female patient at 21+0 weeks of gestation, BMI 27.4 kg/m^2) fetal heart processed by fetal intelligent navigation echocardiography (FINE). 1. three vessel-trachea view; 2. four-chamber view; 3. five-chamber view; 4. left ventricular outflow tract; 5. short axis view of great vessels/right ventricular outflow tract; 6. abdomen/stomach; 7. ductal arch; 8. aortic arch; and 9. superior and inferior vena cava.

The acquired volumes have been examined by the same physicians either onsite on the ultrasound machine using the installed FINE software or offsite on a personal computer using the same software. FINE generates nine standard fetal echocardiography planes (1. four-chamber view; 2. five-chamber view; 3. left ventricular outflow tract; 4. short-axis view of great vessels/right ventricular outflow tract; 5. three vessel-trachea view; 6. abdomen/stomach; 7. ductal arch; 8. aortic arch; and 9. superior and inferior vena cava). To generate these planes, the software instructs the user to mark seven anatomical landmarks of the fetal heart (1. cross-section of the aorta at the stomach level; 2. cross-section of the aorta at the level of the four-chamber view; 3. crux; 4. right atrial wall; 5. pulmonary valve; 6. cross-section of the superior vena cava; and 7. transverse aortic arch). The program gives specific hints to ensure the best result (e.g., written instructions, pictures of the landmarks, and automatic presentation of the correct planes). The nine echocardiographic views of the normal and abnormal fetal heart created by FINE are shown in Figure 1. In addition, the whole process of generating the views can be found in Video 1.

**Video 1:** Fetal Intelligent Navigation Echocardiography (FINE) Screen Video. Showing the creation of all views of the structurally normal fetal heart by using FINE.

Rating by an expert panel

All planes reconstructed by the FINE software were rated by an expert panel regarding proper reconstruction. Image quality was evaluated for all volumes ranging from "very good" to "poor".

Statistics

The data were investigated regarding many properly reconstructed planes. GraphPad Prism 9 for Mac (Version 9.51, GraphPad Software Inc., La Jolla, CA, USA), GraphPad QuickCalcs (GraphPad Software Inc., La Jolla, CA, USA), and Microsoft Excel for Mac (Version 16.71, Microsoft Corp., Redmond, WA, USA) were used. Descriptive statistics, t-tests, and McNemar-tests were performed. A statistical level of p < 0.05 was assumed to be significant.

## Results

In total, 328 twins underwent STIC-volume acquisition. We excluded 20 cases, of which 12 were first-trimester fetuses, and 8 had abnormal hearts. Three hundred eight fetuses were conducted to conclude the analysis. The mean gestational age (GA) was 22.1 weeks (14.0 to 32.9 weeks), and the mean BMI at the scanning date was 27.3 kg/m^2^ (19.2 to 40.7 kg/m^2^). Concerning chorionicity, 55.8% (n = 86) of the pregnancies were dichorial, and 44.2% (n = 68) were monochorial. The cases were rated regarding image quality. 2.7% (n = 8) stated "very good", 57.4% (n = 173) stated "good", 37.2% (n = 112) were "moderate," and 2.7 (n = 8) were "poor" quality. In 7 cases, no sufficient STIC volume could be obtained. STIC-volume acquisition in twin 1 was successful in 100% of cases and twin 2 in 95.5%. The failed cases and their corresponding twins were excluded from further analysis.

**Figure 2:**
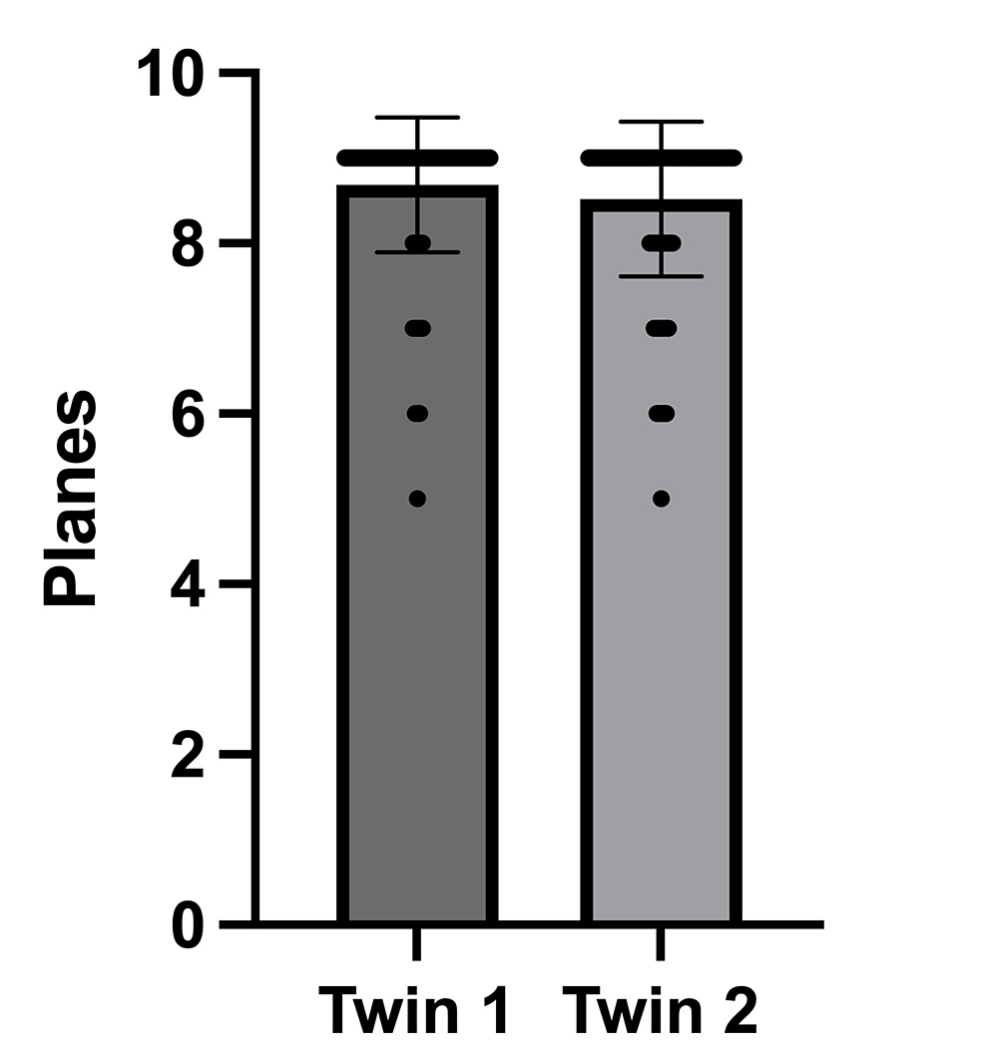
Correctly shown planes after processing with fetal intelligent navigation echocardiography (FINE) [number of planes, mean with SD].

Two hundred ninety-four fetuses (147 pregnancies) remained for further analysis. Regarding the overall depiction rate, FINE properly reconstructed 96.5% of the planes for twin 1, while it reconstructed 94.7% for twin 2 (p = 0.0849, not significant). In 95.9% (twin 1) and 93.9% (twin 2), at least 7 planes were reconstructed properly (p = 0.6056, not significant). In 89,8% (n = 132) of the pregnancies, at least 7 planes were reconstructed properly for both fetuses. The depiction rates are shown in Table 1 and Figure 2.

**Table 1:** Correctly shown planes after processing with fetal intelligent navigation echocardiography (FINE) [percent].

Group	Overall depiction rate [% of all planes]	p-value
Twin 1	96.5	0.0849
Twin 2	94.7	
Group	Depiction rate >= 7 planes [% of all cases]	p-value
Twin 1	95.9	0.6056
Twin 2	93.9	

The sagittal planes showed the highest drop-out rates. The drop-out rates were 8.8% and 15.0% (twin 1 and twin 2) for the ductal arch, 8.8% and 12.9% for the aortic arch, and 6.8% and 7.5% for the superior and inferior vena cava, respectively. The drop-out rates for all planes are shown in Table 2.

**Table 2:** Drop-out rate per view after processing with fetal intelligent navigation echocardiography (FINE) [percent].

Plane	Twin 1 drop-out rate [%]	Twin 2 drop-out rate [%]
three vessel-trachea view	1.4	0.7
four-chamber view	0.0	0.0
five-chamber view	1.4	2.7
left ventricular outflow tract	1.4	2.7
right ventricular outflow tract	2.7	5.4
abdomen	0.0	0.7
ductal arch	8.8	15.0
aortic arch	8.8	12.9
vena cava	6.8	7.5

## Discussion

The present study provides exciting results regarding fetal echocardiography and FINE in twin pregnancies. First, FINE demonstrated its reliability in the present study sample. The overall depiction rates of the cardiac planes were 96.5% (twin 1) and 94.7% (twin 2). In 95.9% (twin 1) and 93.9% (twin 2), at least 7 cardiac planes were correctly reconstructed. These results are consistent with those derived from previous studies [11,23,24,30,34,35]. In our past studies, FINE showed overall depiction rates between 83.3% and 96.9%. As shown in the other literature mentioned, FINE reconstructed the cardiac planes up to 100%.

Second, the present study is, as far as we know, the first one to evaluate the feasibility and performance of FINE in twin pregnancies. In our study, no significant differences between twin 1 and twin 2 could be detected concerning the correct depiction of the diagnostic planes. FINE being used in twin pregnancies might be helpful to improve medical care in those patients. The longer duration of the conventional fetal echocardiography [13] and the greater risk of fetal cardiac defects, especially in monochorionic twins [14,16] - our study included 44.2% monochorionic pregnancies - could allow technical solutions like FINE to aid as well in time reduction as in increasing detection rates. This might be helpful to establish the technique for screening purposes in daily routine. 

Diagnosing CHD prenatally is difficult, and conventional screening programs show rates ranging from only 15-39% [9,10]. The relatively low detection rates in the low-risk population not undergoing specialized ultrasound remain challenging. Technical solutions could help overcome the current problems. Several papers are dealing with FINE in cases of CHD. A sensitivity of 98% and a specificity of 93% using FINE in cases of CHD were reported [31]. According to their work, the diagnosis based on FINE suited the final diagnosis in 74% of cases [31].

Furthermore, the ability of the software to prenatally detect dextrocardia with complex CHD and tetralogy of Fallot (TOF) with pulmonary atresia was demonstrated, too [25,36]. Another group used FINE in fetuses with d-transposition of the great arteries (D-TGA) and reported an 85.7% success rate in generating two or three specific abnormal cardiac views [32]. They concluded that FINE could be promoted as a screening tool for this specific CHD. Moreover, another paper pointed out comparable results and conclusions in 25 cases of the double‐outlet right ventricle (DORV) [37].

In recent years, different features of FINE and the application of this software in special situations have been investigated. Our group recently tested the so-called FINE static mode in 257 cases. The static mode allows the automatic generation of the nine diagnostic planes using fast-acquired STIC volumes (e.g., 1s). The FINE standard mode reconstructed at least 7 planes in 96.9%, while the FINE static mode did so in 94.2% [35]. According to that, we concluded the possible usefulness of the FINE static mode in moving fetuses. Another of our recent works investigated FINE used by investigators with different experience levels in fetal echocardiography. All investigators generated the diagnostic planes quickly using FINE (21-74 seconds per case) [30].

Furthermore, we demonstrated the accuracy of FINE in fetuses with inconvenient spine positions [34]. In the optimal fetal spine position between 5 and 7 o'clock, 94.9% of the diagnostic planes were displayed properly, whereas the depiction rates in the other groups ranged from 92.4% to 87.3% [34]. The rates were significantly lower in suboptimal positions but did not fall below 85%. When promoting technical solutions as a perspective for screening, a depiction rate of at least 85%, even in inconvenient fetal positions, is helpful. In our eyes, STIC volumes processed by FINE might aid untrained examiners in detecting and characterizing CHD cases. It must be said, however, that although FINE contains some automatic alarms (e.g., the Breech Alert, which notifies the user that the fetus appears to be in a breech presentation, and the Spine Location Alert, which notifies the user that the fetal spine appears to be located at a position that is different from what is recommended), it does not currently specifically alert the user to the possible presence of a heart defect. This is a distinct limitation of FINE that may or may not be fixed in the future.

Another group used Doppler combined with FINE, demonstrating promising results [38]. The rate of successful generation of eight or more fetal echocardiography views with precise Doppler information was 89-100% [38]. In addition, That work included four cases of CHD, in which color Doppler FINE demonstrated abnormal fetal cardiac anatomy or hemodynamic flow [38]. Using color Doppler FINE allowed the accurate diagnosis of the fetal hypoplastic left heart (HLHS) and coarctation of the aorta (CoA) at 26 weeks of gestation [39].

Roberts described two ways in which 4D echocardiography could be helpful to enhance the identification of CHD: First, local recording of STIC volumes at one site following remote examination by an expert in fetal echocardiography at another site; second, acquisition, storage, and investigation of the data by the same investigator later on [40]. He proposed that a general screening program using STIC technology would have to work as a hybrid of the models described above based on the individual risk for CHD [40]. The usefulness of volumetric echocardiography is often only sometimes accepted by other authors. For example, the lacking standardization and high user dependency are criticized. The success rates of obtaining STIC volumes in normal fetal hearts range widely from 26 to 100% [12,17,19,23,41-43]. Our present study ranged from 100% for twin 1 to 95.5% for twin 2. Another group demonstrated successful STIC acquisition in 97.3% of cases, but all necessary planes could be obtained in just 49% [44]. They concluded that STIC might be used to improve the detection of CHD, but the experience of the user remains the most critical factor for adequate quality [44].

One of the main reasons for the low detection rates of CHD in a general screening is the lacking skill of the investigator. Our work indicates that FINE performs well in inexperienced hands [30]. To increase the prenatal detection rates of CHD, investigators might learn to obtain and analyze a sufficient STIC volume with FINE easier than performing an accurate conventional 2D fetal echocardiography. In our eyes, software tools are promising because, despite several guidelines and training programs, the detection of CHD in a general screening setting remains low. Following the guidelines of the ISUOG, the variation in the detection rates is mostly due to differences in investigator expertise [29]. To improve a sonographic screening program, long and hard training is necessary. For example, the detection rate of major cardiac anomalies increased after implementing an intense 2-year training program in England [45]. More operator-independent methods are promising in raising identification rates and improving time- and cost-effectiveness.

We have strengths and limitations. The STIC volumes used for the study have been obtained under real-life conditions (e.g., not selecting by BMI or fetal position) and might therefore be representative for other sites. In addition, we did not restrict ourselves to the ideal time frame for fetal echocardiography. We included a relatively large sample size with a broadly defined gestational age, which has been validated in previous studies. The potential usefulness of FINE in the first trimester must still be investigated. Our volumes were collected and analyzed by experts in their field, so the data quality is high. The retrospective design of our study is a limitation. We might have had a selection bias because not all patients presenting within the study period were scanned with 3D/4D ultrasound.

## Conclusions

Our results indicate that FINE in twin pregnancies is a reliable method. No significant difference between the depiction rates of twin 1 and twin 2 could be detected. Beyond that, the depiction rates are as high as those derived from singleton pregnancies. Due to the challenges of fetal echocardiography in twin and multiple pregnancies (i.e., greater rates of cardiac anomaly and more difficult scans), FINE might be a tool to improve the quality of medical care in those pregnancies. Our supporting material indicates that FINE might as well work in triplet pregnancies. FINE can support examining the fetal heart in a standardized and time-effective manner. Investigation of the fetal heart by FINE might raise the detection rates of CHD during general screening. Future studies should be conducted to investigate the application of FINE in a general screening setting and the first trimester of pregnancy.
